# Supplementary material for: Complete avian malaria parasite genomes reveal features associated with lineage-specific evolution in birds and mammals
Source: Genome Res. 2018 Apr;28(4):547–60. doi: 10.1101/gr.218123.116 (PMC5880244; doi:10.1101/gr.218123.116)
Supplement: Supplemental Material [file supp_28_4_547__index.html]

Complete avian malaria parasite genomes reveal features associated with lineage-specific evolution in birds and mammals — Supplemental Material 

# Complete avian malaria parasite genomes reveal features associated with lineage-specific evolution in birds and mammals

## Supplemental Material

- Supplemental\_material\_GENOME\_2016\_218123.pdf
- Supplemental\_Table\_S6.xlsx
- Supplemental\_Table\_S7.xlsx
- Supplemental\_Table\_S8.xlsx
- Supplemental\_Table\_S9.xlsx
- Supplemental\_Table\_S10.xlsx
