# Supplementary material for: Complete avian malaria parasite genomes reveal features associated with lineage-specific evolution in birds and mammals
Source: Genome Res. 2018 Apr;28(4):547–60. doi: 10.1101/gr.218123.116 (PMC5880244; doi:10.1101/gr.218123.116)
Supplement: Supplemental Material [file supp_gr.218123.116_Supplemental_material_GENOME_2016_218123.pdf]

Complete avian malaria parasite genomes reveal features associated with lineage specific evolution in birds and mammals

Supplemental Figures, Tables and Methods

Ulrike Böhme[1]*, Thomas D. Otto[1]*, James Cotton[1], Sascha Steinbiss[1], Mandy Sanders[1], Samuel O. Oyola[1,2], Antoine Nicot[3], Sylvain Gandon[3], Kailash P. Patra[4], Colin Herd[1], Ellen Bushell[1], Katarzyna K. Modrzynska[1], Oliver Billker[1], Joseph M. Vinetz[4], Ana Rivero[5], Chris I. Newbold[6], Matthew Berriman[1]

* These authors contributed equally to this work.

Supplemental Figures 3

Supplemental Figure S1. Conservation of synteny in the core regions of chromosomes. 3

Supplemental Figure S2. Unrooted phylogeny of *Apicomplexan* parasites. 4

Supplemental Figure S3. Results of alternative phylogenetic analyses. 5

Supplemental Figure S4. Comparisons of *P. knowlesi*, *P. relictum* , *P. gallinaceum* and *P. falciparum* 3D7 reveal avian malaria-specific genes 6

Supplemental Figure S5. 3D modeling of Ku70. 7

Supplemental Figure S6. Differentially distributed pseudogenes between *P. relictum* and *P. gallinaceum.* 8

Supplemental Figure S7. Comparisons of *P. knowlesi*, *P. relictum*, *P. gallinaceum* and *P. falciparum* 3D7 redefine lineage specific distributions 9

Supplemental Figure S8. Shikimate Pathway highlighting differences in the avian malaria genomes. 10

Supplemental Figure S9. Organization of subtelomeric regions. 11

Supplemental Figure S10. Core retrotransposon fragments. 12

Supplemental Figure S11. Core retrotransposon of *P. relictum*. 13

Supplemental Figure S12. Maximum likelihood tree of avian malaria retrotransposons. 14

Supplemental Figure S13. Subtelomeric gene families in *P. gallinaceum* and *P. relictum.* 15

Supplemental Figure S14. Overview of gene families. 16

Supplemental Figure S15. Comparison of Reticulocyte binding proteins (RPB) Meme motifs comparison. 17

Supplemental Tables 18

Supplemental Table S1. Core genes only present in *P. gallinaceum* 8A, *P. relictum* SGS1. 18

Supplemental Table S2. Core genes that appear to be restricted to *the* Laverania subgenus (*P. falciparum* 3D7, *P. reichenowi* CDC) and the avian clade (*P. gallinaceum*, *P. relictum*) 20

Supplemental Table S3. Core genes specific to *P. knowlesi/P. vivax/P. malariae/P. ovale* clade and *P. gallinaceum, P. relictum*. 21

Supplemental Table S4. Core genes pseudogenized in *P. gallinaceum* and/or *P. relictum* 22

Supplemental Table S5. Core genes missing in *P. gallinaceum* and *P. relictum* 23

Supplemental Table S6. Multigene families in the genome of *P. gallinaceum* and *P. relictum*. . 24

Supplemental Table S7. Gene summary of *P. gallincaeum* 24

Supplemental Table S8. d_N/d_S values for all genes with 1:1 orthologous across six species. 24

Supplemental Table S9. Top 250 d_N/d_S values from pairwise comparisons within three lineages.24

Supplemental Table S10. d_N values for all genes with 1:1 orthologous across eight species. 24

Supplemental Methods.. 25

Relationship between *Plasmodium* species 25

Whole genome sequencing of *P. gallinaceum* 25

Collection of genomic DNA from *P. relictum*.. 25

Genome assembly of *P. relictum*.. 26

Phylogenetic analysis 26

Dating analysis.. 27

Supplemental References 28

Supplemental Figures

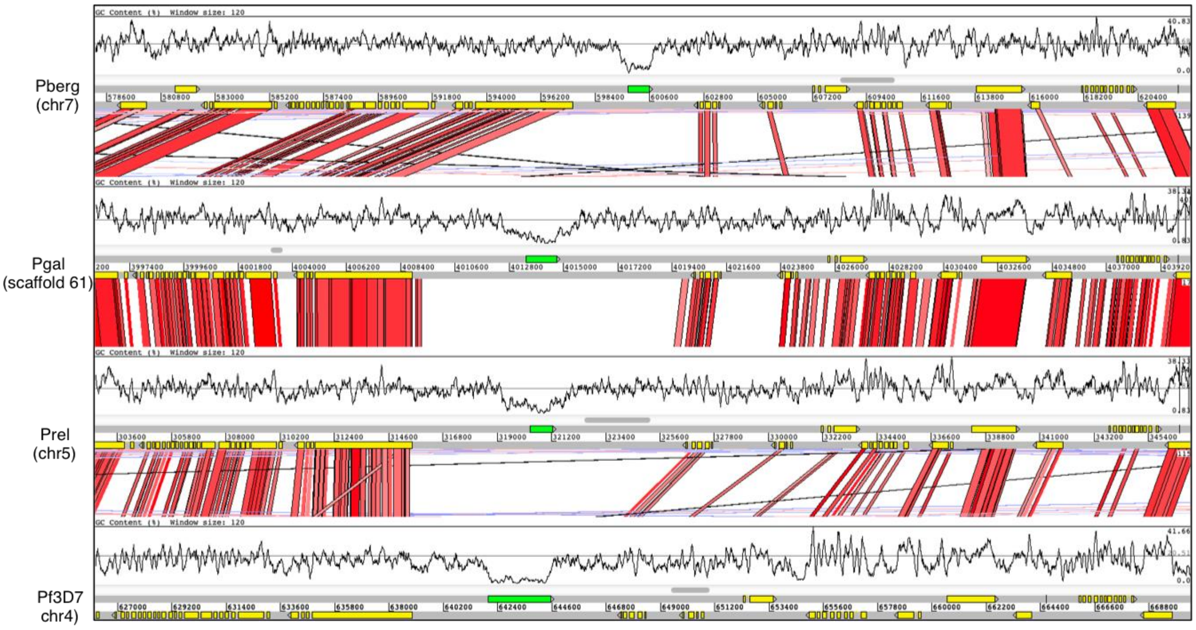

Supplemental Figure S1. Conservation of synteny in the core regions of chromosomes.
ACT (Artemis Comparison Tool) screenshot showing a comparison of centromere-proximal regions of an illustrative chromosome in *P. berghei* ANKA (Pberg) (chromosome 7), *P. gallinaceum* (Pgal) (scaffold 61)*, P. relictum* (Prel) (chromosome 5) and *P. falciparum* 3D7 (Pf3D7) (chromosome 4). This conservation of synteny in the core regions is representative for all other *Plasmodium* chromosomes. The red blocks represent sequence similarity (TBLASTX). The centromere is shown in green. Yellow coloured boxes represent genes. The graph shows the GC-content, the highest GC content is 40%, the GC content at the centromere is 3%.

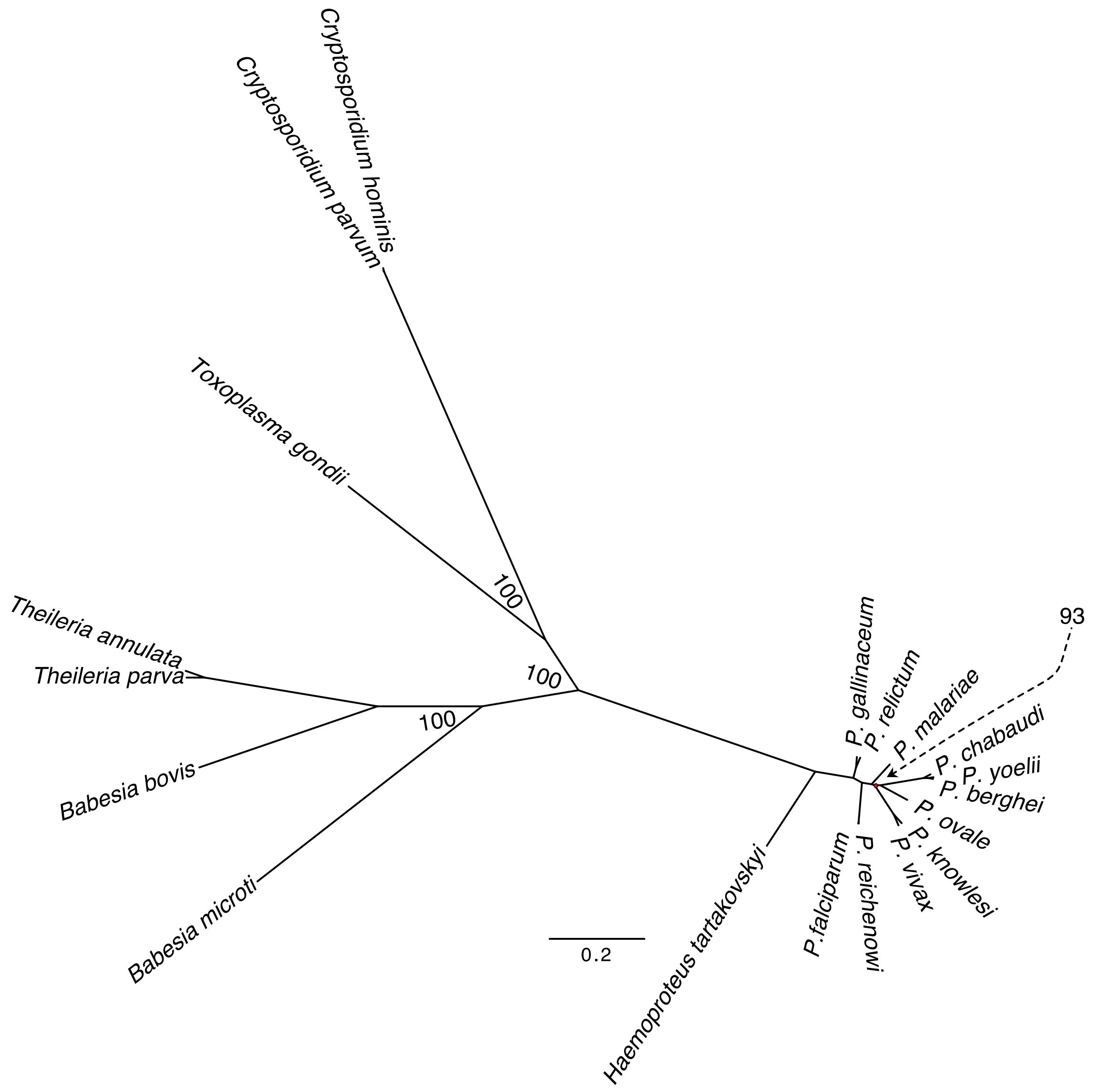

Supplemental Figure S2. Unrooted phylogeny of *Apicomplexan* parasites.

Unrooted phylogeny of *Apicomplexan* parasites, based on 879 single-copy orthologues, showing full results of the partitioned maximum likelihood analysis shown in Figure 2. All nodes are supported by 100 out of 100 bootstrap samples except the marked node, where support level is shown with an arrow.

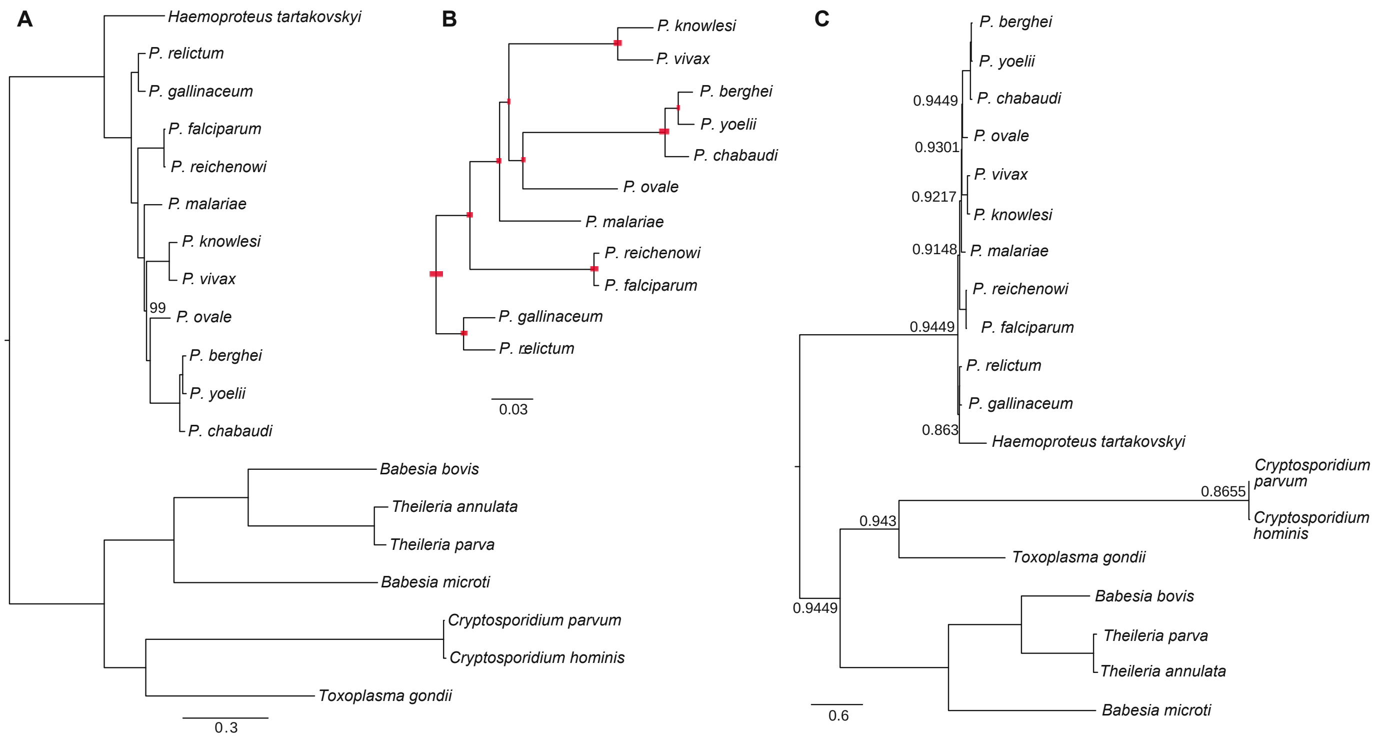

Supplemental Figure S3. Results of alternative phylogenetic analyses.

(A) Maximum-likelihood phylogeny with a non-partitioned analysis under the LG4X model. Bootstrap support values were 100 except for node indicated. (B) Bayesian phylogeny of *Plasmodium spp.* rooted with *Haemoproteus tartakovskyi* calculated under CAT-Poisson model. All nodes had a posterior probability of 1.0; salmon pink boxes on nodes indicate uncertainty (95% highest posterior density confidence intervals) in the length of the branch below that node. (C) Majority-rule consensus of posterior samples of tree topology from Bayesian analysis under CAT-Poisson model. Nodes had posterior probability of 1.0 except where indicated.

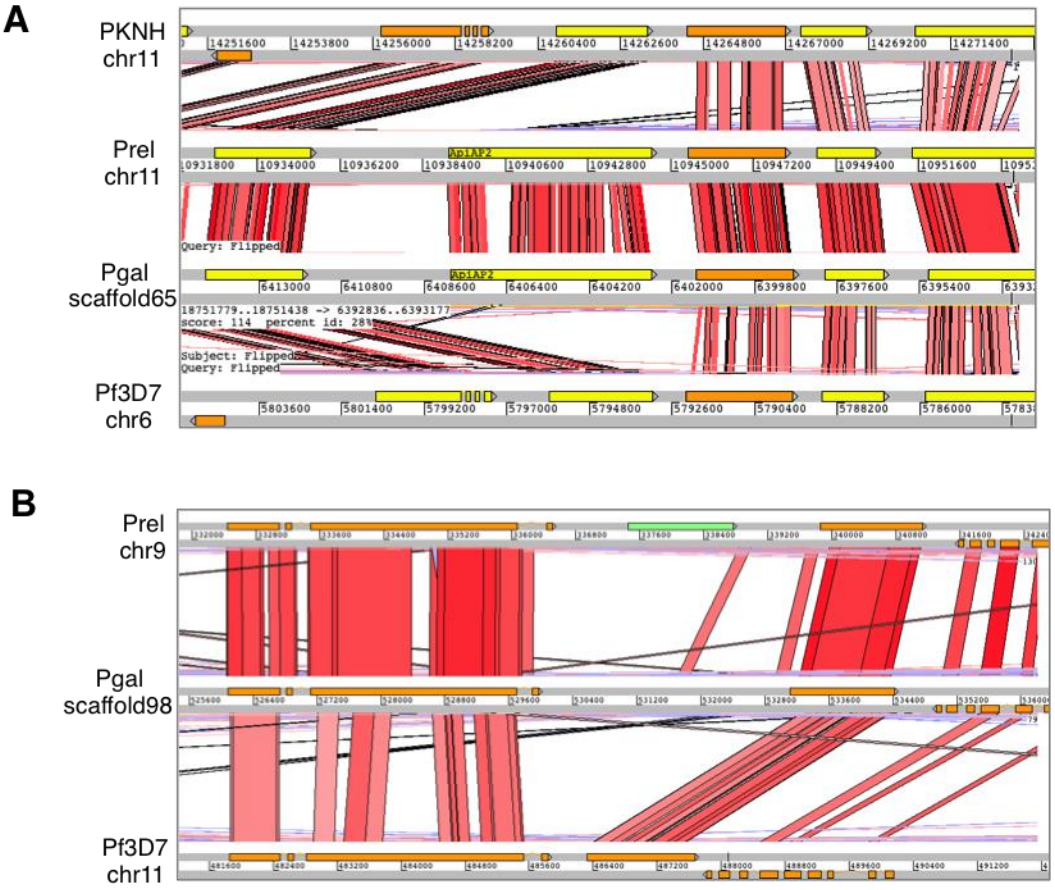

Supplemental Figure S4. Comparisons of *P. knowlesi*, *P. relictum* , *P. gallinaceum* and *P. falciparum* 3D7 reveal avian malaria-specific genes

Screenshots of comparisons using ACT that show (A) an avian malaria specific ApiAP2 protein (PGAL8A_00142800, PRELSG_113400) and (B) a gene of unknown function (green) within the core region of a chromosome that is only present in *P. relictum* (PRELSG_0909800). Grey bars represent the forward and reverse DNA strands. The red blocks between sequences represent sequence similarity (TBLASTX). PKNH, *P. knowlesi*; Prel, *P. relictum*; Pgal, *P. gallinaceum*; and Pf3D7, *P. falciparum* 3D7.

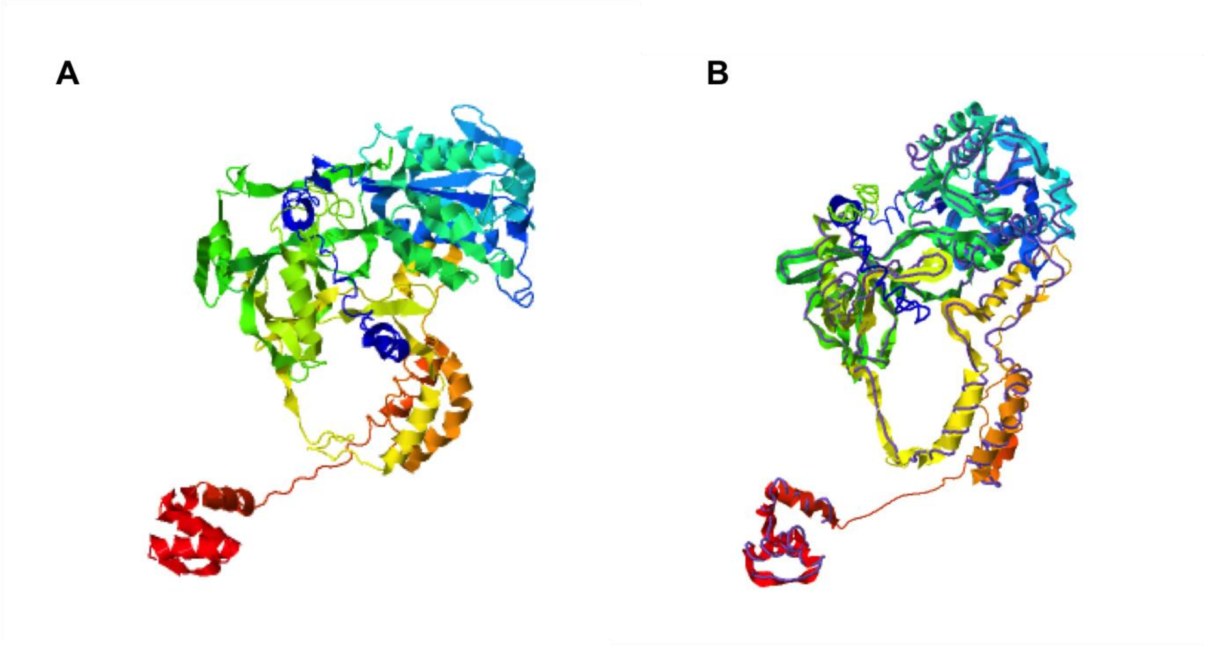

Supplemental Figure S5. 3D modeling of Ku70.

(A) A three-dimensional model of Ku70 (PGAL8A_000014200) created using I-TASSER (Yang et al. 2015). The structure is visualized as a rainbow cartoon by the JSmol applet. (B) Ku70 (PGAL8A_000014200) structure is shown in cartoon format, while the highest scoring identified structural analog in PDB (1JEQ, Ku heterodimer, *homo sapiens*) is displayed using backbone trace.

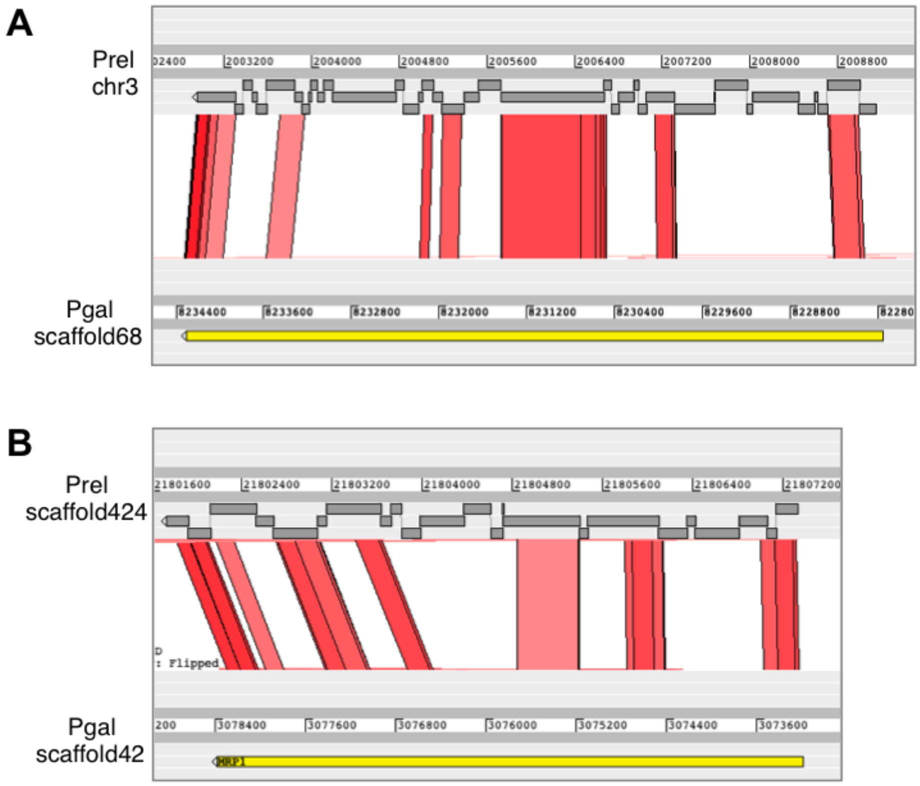

Supplemental Figure S6. Differentially distributed pseudogenes between *P. relictum* and *P. gallinaceum.*

Screenshots from ACT showing: (A) An avian-malaria specific gene in a core region of a chromosome and encoding a protein kinase. The kinase is a pseudogene in *P. relictum* (PRELSG_0314000) but a functional gene in *P. gallinaceum* (PGAL8A_00181200). (B) A degenerate multidrug-resistance associated protein 1, MRP1 (PRELSG_0028300) in *P. relictum*. There is an additional MRP1 (PRELSG_1445800) in *P. relictum* that is not pseudogenised. The red blocks between sequences represent sequence similarity (TBLASTX). Coloured boxes represent genes. Pseudogenes in *P. relictum* are shown in grey with multiple frameshifts.

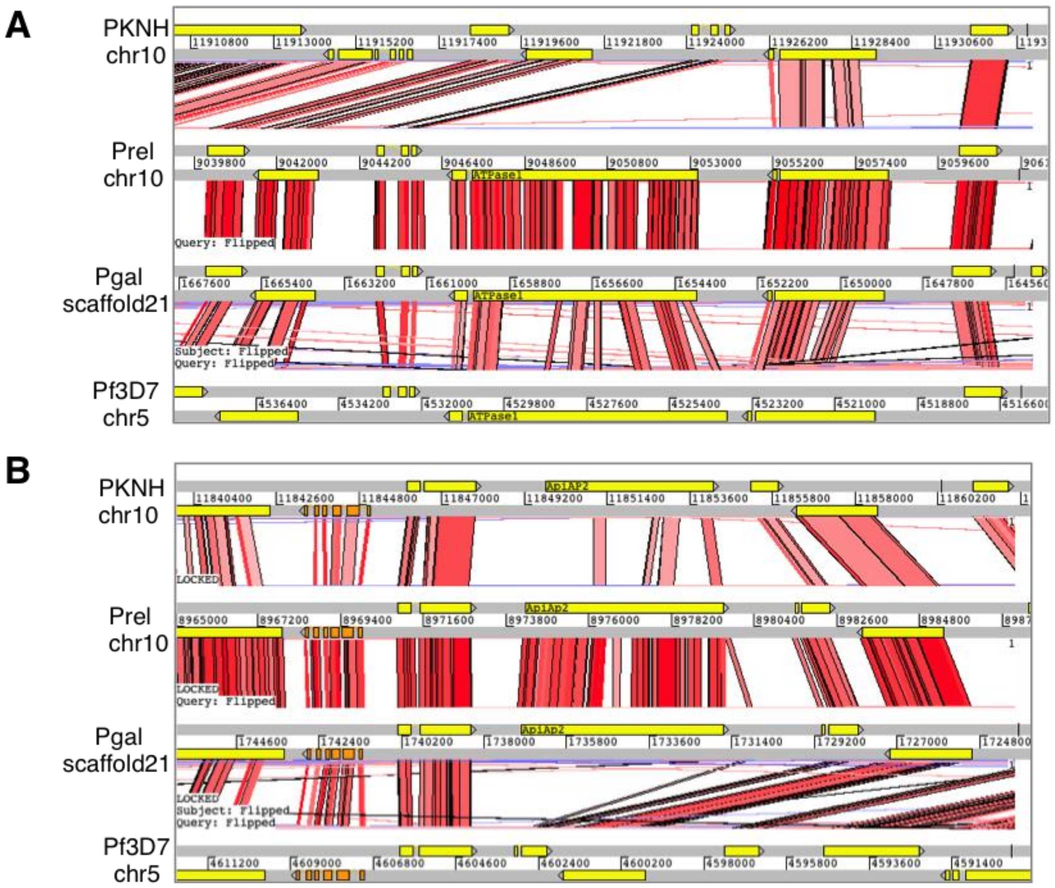

Supplemental Figure S7. Comparisons of *P. knowlesi*, *P. relictum*, *P. gallinaceum* and *P. falciparum* 3D7 redefine lineage specific distributions.

Screenshot of comparisons using ACT that show (A) an ATPase1 that was thought to be Laverania-specific (PF3D7_0516100) but is present in *P. gallinaceum* (PGAL8A_00025200) and *P. relictum* (PRELSG_1015800). (B) An ApiAP2 protein (PKNH_1015400) that was thought to be specific to the knowlesi/vivax/cynomolgi clade but is also present in *P. gallinaceum* (PGAL8A_00039400) and *P. relictum* (PRELSG_1014000). PKNH, *P. knowlesi*; Prel, *P. relictum*; Pgal, *P. gallinaceum*; and Pf3D7, *P. falciparum* 3D7. The red blocks between sequences represent sequence similarity (TBLASTX). Coloured boxes represent genes.

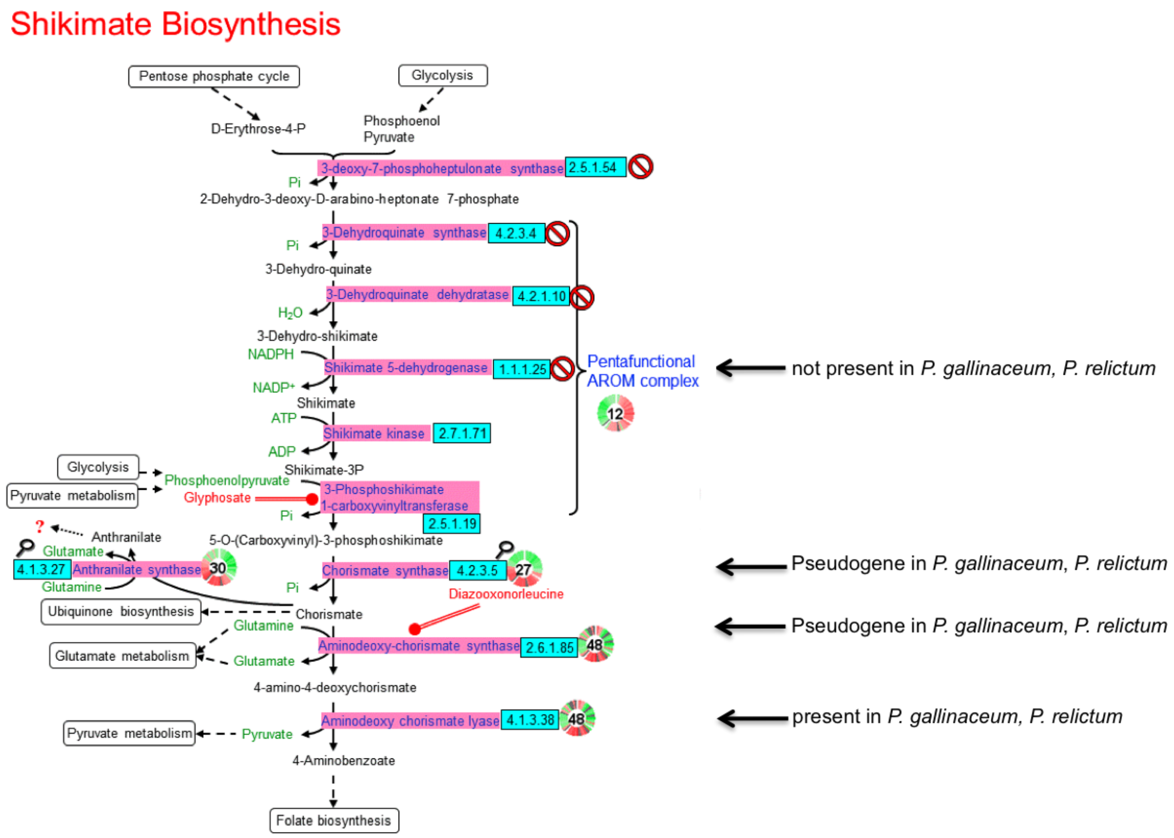

Supplemental Figure S8. Shikimate Pathway highlighting differences in the avian malaria genomes. Chorismate synthase (PGAL8A_00151500, PRELSG_1125300) and aminodeoxy-chorismate synthase (PGAL8A_00435300, PRELSG_0720000) are pseudogenised in *P. gallinaceum* and *P. relictum*. The pentafunctional AROM polypeptide is missing in *P. gallinaceum* and *P. relictum* (orthologue in *P. falciparum* is PF3D7_0206300). The metabolic pathway illustration is from the Malaria Parasite Metabolic Pathway site (http://mpmp.huji.ac.il; Ginsburg and Abdel-Haleem 2016).

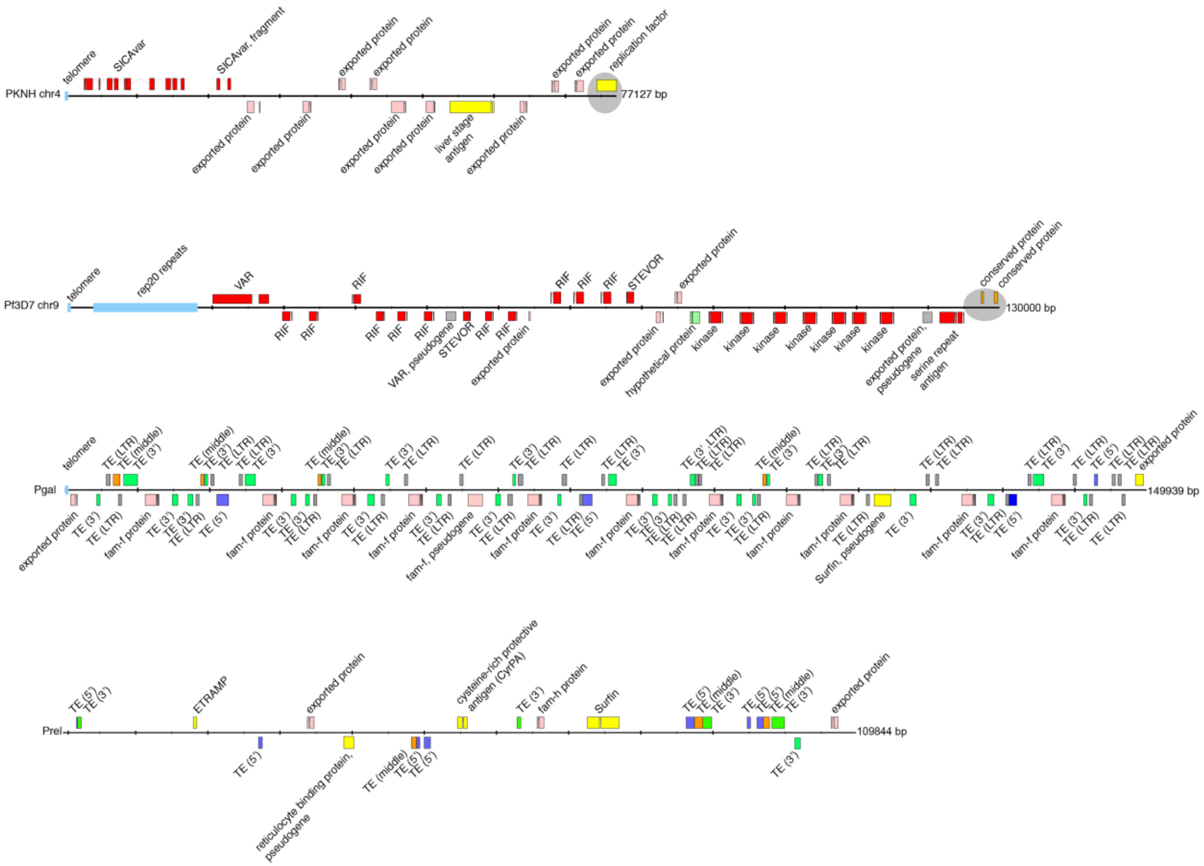

Supplemental Figure S9. Organization of subtelomeric regions.

Organization of subtelomeric regions of chromosomes 4 of *P. knowlesi* (left region), 9 of *P. falciparum* 3D7 (left region), *P. gallinaceum* (scaffold 70) and *P. relictum* (scaffold 174). The order and orientation of the genes are shown. Exons are shown in coloured boxes with introns as linking lines. As a comparison, a subtelomeric region of *P. falciparum* 3D7 chromosome 9 and *P. knowlesi* chromosome 4 is shown. The shaded/grey areas mark the start of the conserved, syntenic regions. Transposable element (TE) (LTR), long terminal repeat; TE (3'), 3'part of TE (includes RNaseH domain and integrase domain); TE (middle) (includes the reverse transcriptase domain); TE (5'), 5'part of TE (includes CCHC-type zinc finger domain and aspartic protease domain).

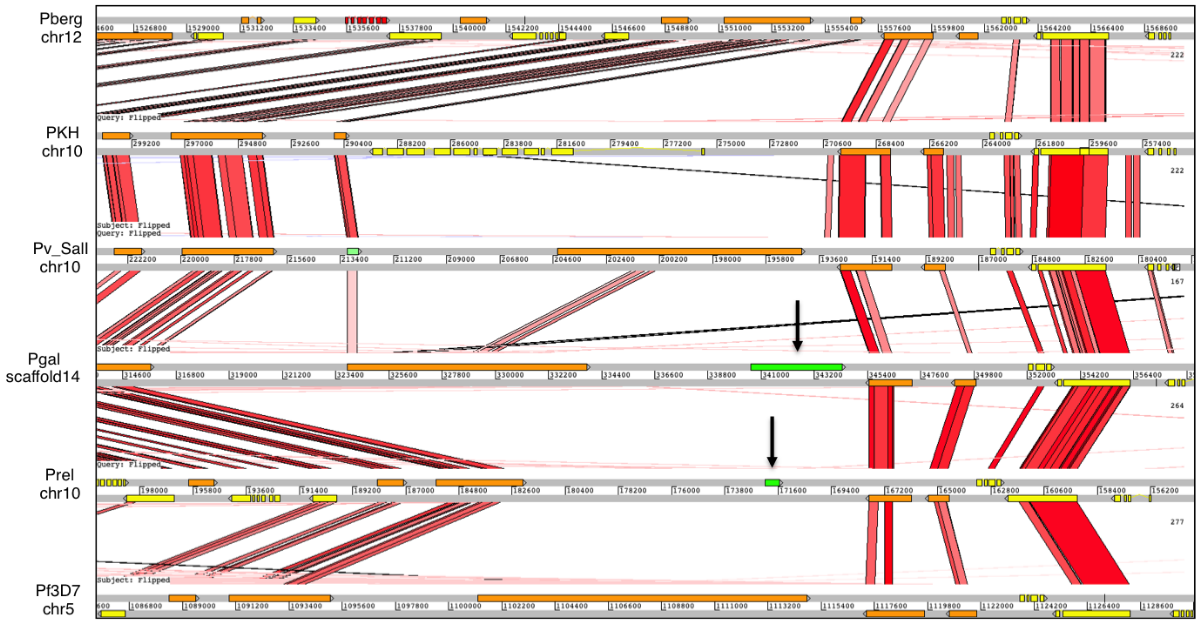

Supplemental Figure S10. Core retrotransposon fragments.

ACT comparison showing the core retrotransposon fragment in *P. gallinaceum* (scaffold 14) and *P. relictum* (chromosome 10) (green with arrow). Pberg, *P. berghei*; PKH, *P. knowlesi*; Pv_Sal1, *P. vivax* Sal1; Pgal, *P. gallinaceum*; Prel, *P. relictum* and Pf3D7, *P. falciparum* 3D7.

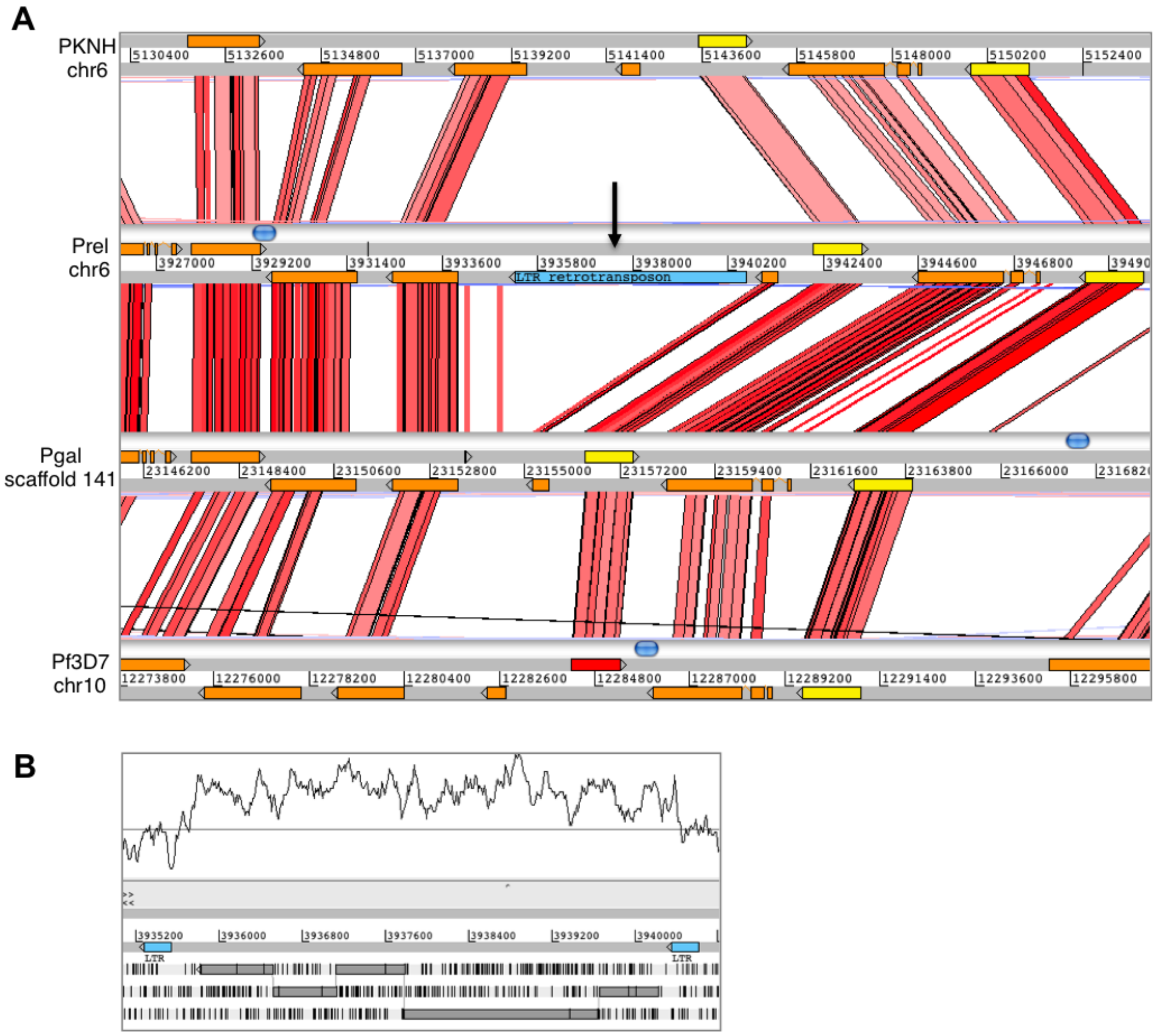

Supplemental Figure S11. Core retrotransposon of *P. relictum*.

(A) Screenshot of an ACT comparison showing a retrotransposon of *P. relictum* (blue with arrow) in the core region of chromosome 6. The gene to the right of the retrotransposon is PRELSG_0613200. (B) A close-up Artemis view of the retrotransposon showing frameshifts and GC content (graph).

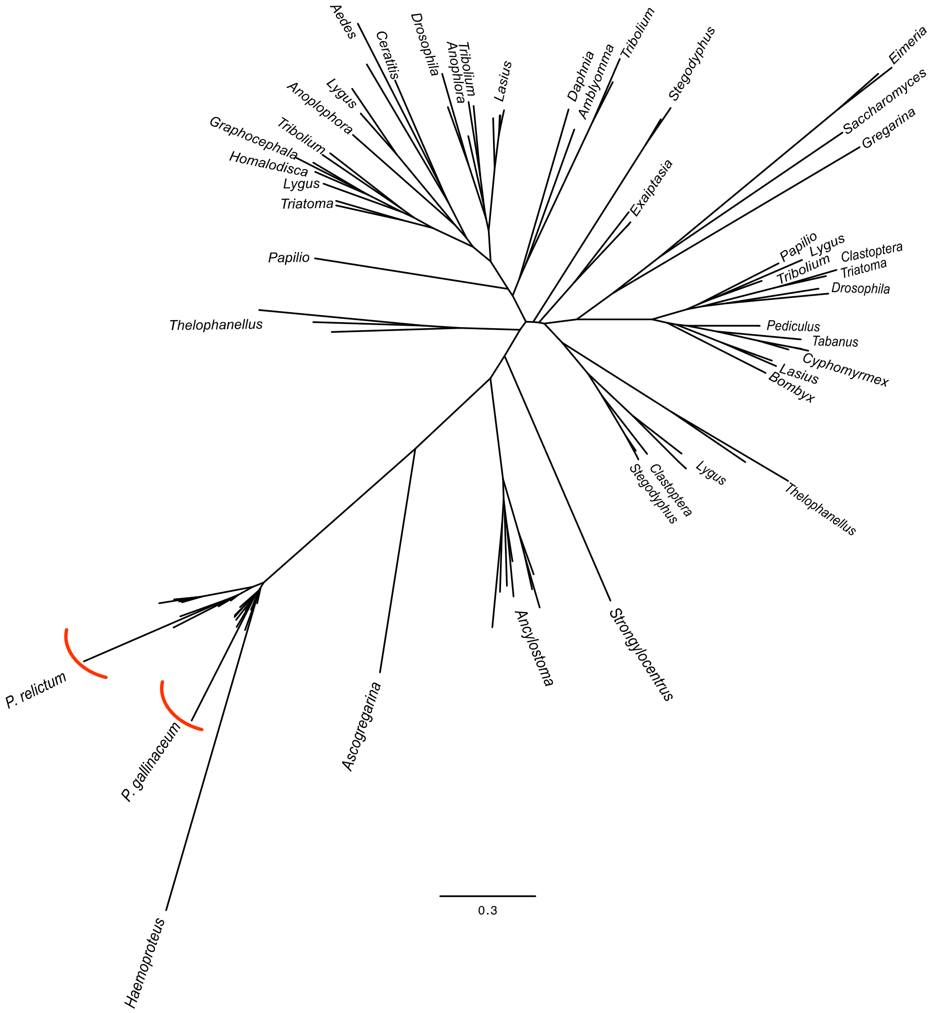

Supplemental Figure S12. Maximum likelihood tree of avian malaria retrotransposons.

Tree showing the differences between *Eimeria* and *P. gallinaceum/P. relictum* transposable elements (TE). It further shows the similarity of the avian TEs to the TEs found in *Haemoproteus tartakovskyi*. Sequences were selected from the top 100 best hits from an NCBI BLAST of the *P. gallinaceum* sequence, plus the *Eimeria* and yeast sequence.

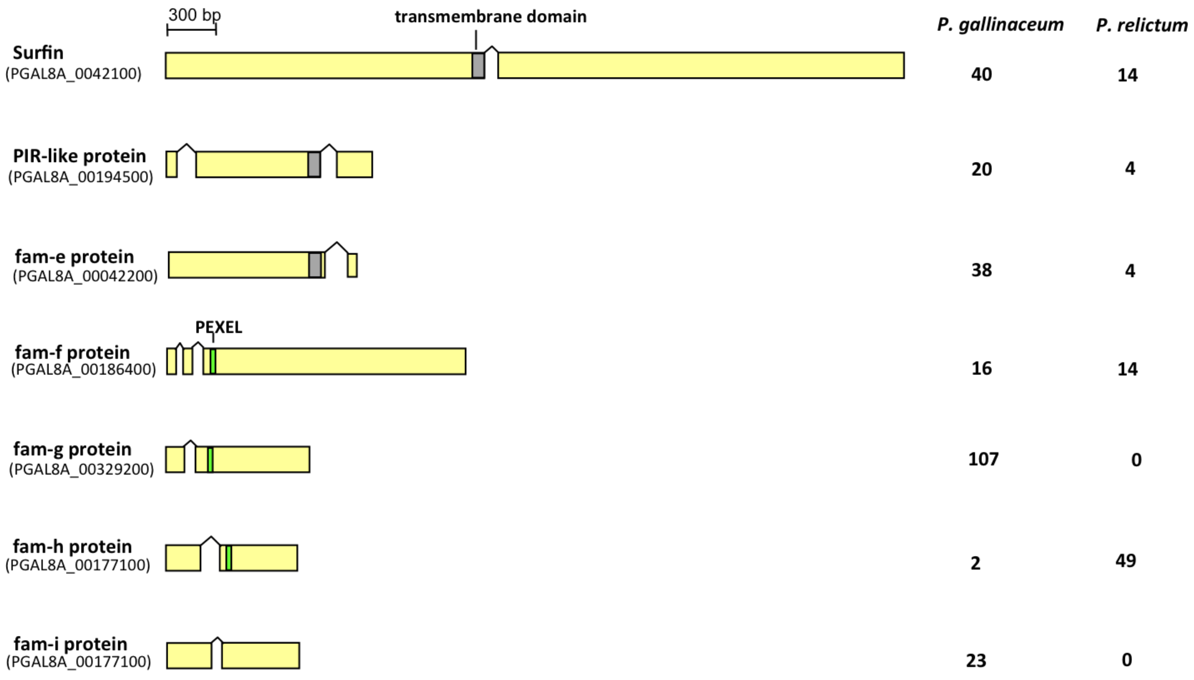

Supplemental Figure S13. Subtelomeric gene families in *P. gallinaceum* and *P. relictum.*
There are 4 avian malaria specific gene families (*fam-e, fam-g, fam-h and fam-i*). Although present in other species as a single copy gene, *fam-f* is expanded in *P. gallinaceum* and *P. relictum*. Surfin and PIR-like proteins are encoded by multigene families present in other *Plasmodium* spp.

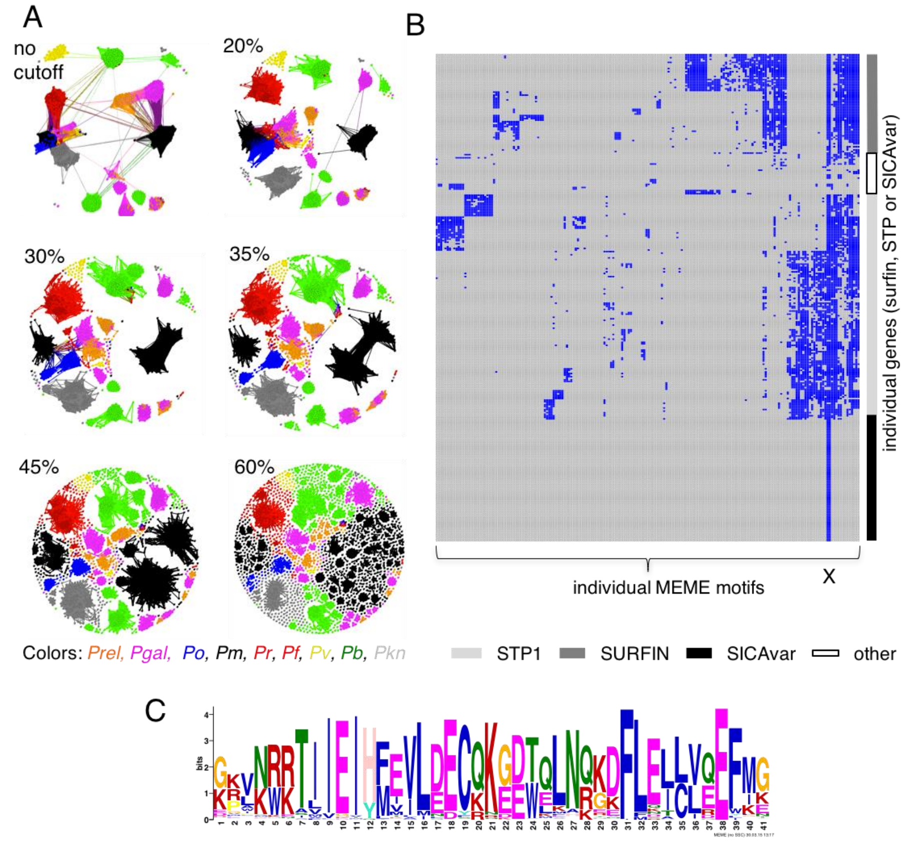

Supplemental Figure S14. Overview of gene families.

(A) The gene network if different cut-off levels (no cutoff, 20%, 30%, 35%, 45% and 60%) (B) The MEME motif occurrence matrix showing all genes that have at least 2 motifs found in STP1 or SURFIN. Two motifs (X) are shared in the majority of the samples. (C) A single motif is shared between all STP1, SURFIN and SICAvar proteins. Prel, *P. relictum*; Pgal, *P. gallinaceum*; Po, *P. ovale*; Pm, *P. malariae*, Pr, *P. reichenowi*; Pf, *P. falciparum*; Pv, *P. vivax*, Pb, *P. berghei* and Pkn, *Pknowlesi*.

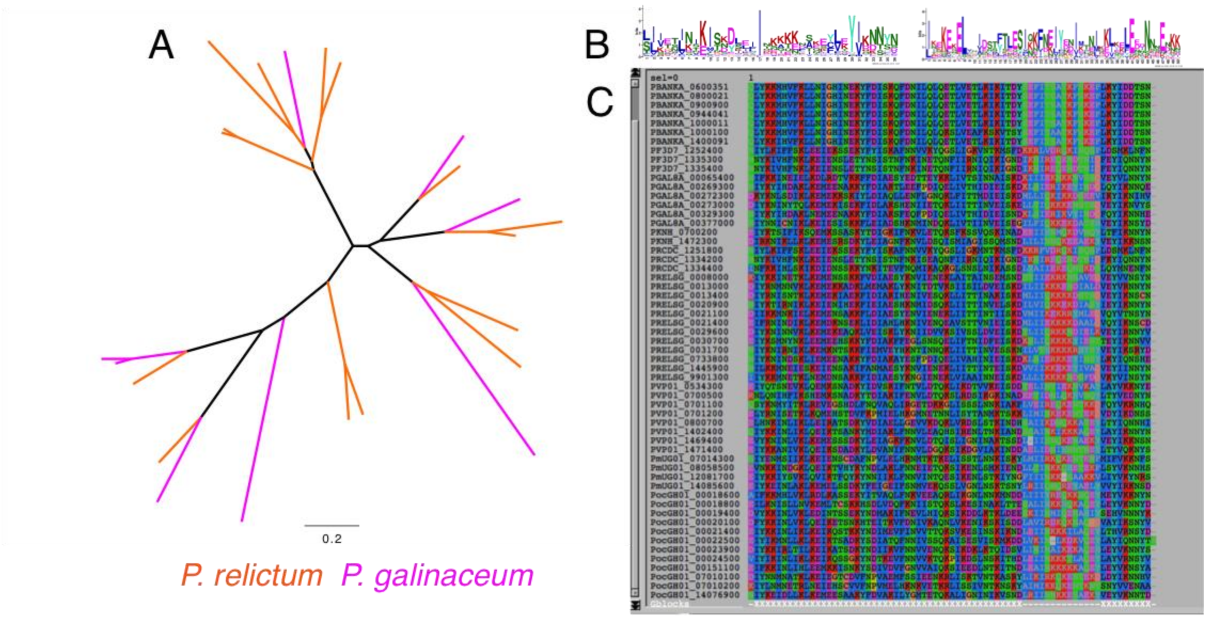

Supplemental Figure S15. Comparison of Reticulocyte binding proteins (RPB) MEME motifs.
(A) Maximum likelihood tree of full length avian RBPs (> 1,500 amino acids). (B) Two MEME motifs of the RBPs (C) Alignment of the two MEME motifs (from panel B) used for the tree in Fig 3, right.

Supplemental Tables

Supplemental Table S1. Core genes only present in *P. gallinaceum* 8A, *P. relictum* SGS1. Pseudogenes are indicated (*).

P. gallinaceum gene	P. *relictum* gene	Protein description
PGAL8A_00014200	PRELSG_0411800	Ku70/Ku80 beta-barrel domain-containing protein, putative
PGAL8A_00017300	PRELSG_0414900	zinc finger protein, putative
PGAL8A_00024500	PRELSG_1029200	cullin, putative
PGAL8A_00033950	PRELSG_1019550	hypothetical protein
PGAL8A_00099100	PRELSG_0214100	AN1-like zinc finger, putative
PGAL8A_00100100	PRELSG_0213100	hypothetical protein
PGAL8A_00101100	PRELSG_0212000	hypothetical protein
PGAL8A_00101200	PRELSG_0211900	hypothetical protein
PGAL8A_00103700	PRELSG_0209400	hypothetical protein
PGAL8A_00114000	PRELSG_0503000	hypothetical protein
PGAL8A_00114300	PRELSG_0502700	ZIP domain-containing zinc transporter, putative
PGAL8A_00121800	PRELSG_1329100	hypothetical protein
PGAL8A_00142800	PRELSG_1134000	transcription factor with AP2 domain(s), putative (ApiAP2)
PGAL8A_00145200	PRELSG_1131600	hypothetical protein
PGAL8A_00155850	PRELSG_1120950	transmembrane protein, putative
PGAL8A_00159300	PRELSG_1117500	SNF1-related protein kinase catalytic subunit alpha, putative (SNF1)
PGAL8A_00161700	PRELSG_1115150*	hypothetical protein
PGAL8A_00165250	PRELSG_1111850	5'-AMP-activated protein kinase subunit beta-1, putative
PGAL8A_00181200	PRELSG_0314000*	protein kinase, putative
PGAL8A_00192400	PRELSG_0516700	serine/threonine protein phosphatase, putative
PGAL8A_00193800	PRELSG_0518100	sodium- and chloride-dependent neutral and basic amino acid transporter, putative
PGAL8A_00195600	PRELSG_1244600*	6-cysteine protein
PGAL8A_00203650	PRELSG_1236550	mitotic checkpoint protein BUB3, putative
PGAL8A_00238300	PRELSG_1201900	glycosyltransferase, putative
PGAL8A_00240200	PRELSG_1268900	hypothetical protein
PGAL8A_00248500	PRELSG_1260400	hypothetical protein
PGAL8A_00276550*	PRELSG_1364400	protein phosphatase 2C, putative
PGAL8A_00280200	PRELSG_1360700	cGMP-specific phosphodiesterase, putative
PGAL8A_00298600	PRELSG_1342200	leucine-zipper-like transcriptional regulator, putative
PGAL8A_00300700	PRELSG_1340150*	hypothetical protein
PGAL8A_00308200	PRELSG_1332600	hypothetical protein
PGAL8A_00320000	PRELSG_1300700	aminotransferase, putative
PGAL8A_00362600	PRELSG_0932600	major facilitator superfamily, putative
PGAL8A_00366900	PRELSG_0936850*	hypothetical protein
PGAL8A_00385000	PRELSG_0806600	peptidase, putative
PGAL8A_00401600	PRELSG_0409100	hypothetical protein
PGAL8A_00407100	PRELSG_0403600	hypothetical protein
PGAL8A_00418750	PRELSG_0703450	NECAP-like protein, putative
PGAL8A_00425900	PRELSG_0710500	hypothetical protein

PGAL8A_00427100	PRELSG_0711700	hypothetical protein
PGAL8A_00444100	PRELSG_0728900*	hypothetical protein
PGAL8A_00456300	PRELSG_1450700	hypothetical protein
PGAL8A_00470100	PRELSG_1464500	amidohydrolase, putative
PGAL8A_00478100	PRELSG_0602500	hypothetical protein
PGAL8A_00481500	PRELSG_0605900	hypothetical protein
PGAL8A_00506400	PRELSG_1408100	hypothetical protein
PGAL8A_00518700	PRELSG_1420200	DnaJ protein, putative
PGAL8A_00521200	PRELSG_1422700	hypothetical protein
PGAL8A_00508700	PRELSG_1410400	hypothetical protein
not present	PRELSG_0909800	hypothetical protein

Supplemental Table S2. **Core genes that appear to be restricted to the *Laveranian* sub-genus (*P. falciparum* 3D7, *P. reichenowi* CDC) and the avian clade (*P. gallinaceum*, *P. relictum*).**

P. gallinaceum	*P. relictum*	*P. falciparum* 3D7	*P. reichenowi* CDC	Protein description
PGAL8A_00012100	PRELSG_0822800	PF3D7_0312900	PRCDC_0312200	hypothetical protein
PGAL8A_00037600	PRELSG_1015800	PF3D7_0516100	PRCDC_0515200	cation-transporting ATPase 1, putative (ATPase1)
PGAL8A_00076500	PRELSG_1002600	PF3D7_0529200	PRCDC_0528300	sugar transporter, putative
PGAL8A_00103400	PRELSG_0209700	PF3D7_0107300	PRCDC_0105200	hypothetical protein
PGAL8A_00135300	PRELSG_1141500	PF3D7_0606800	PRCDC_0605400	hypothetical protein
PGAL8A_00077500	PRELSG_0116600	PF3D7_0801400	PRCDC_0801000	hypothetical protein
PGAL8A_00419400	PRELSG_0704100	PF3D7_0906800	PRCDC_0904900	hypothetical protein
PGAL8A_00356700	PRELSG_0927000	PF3D7_1129850	PRCDC_1128250	UNC-50 protein, putative
PGAL8A_00380300	PRELSG_0801900	PF3D7_1004100	PRCDC_1003500	hypothetical protein
PGAL8A_00351500	PRELSG_0921700	not present	PRCDC_1123300	hypothetical protein
PGAL8A_00509000	PRELSG_1410650	PF3D7_1312700	PRCDC_1311700	hypothetical protein
PGAL8A_00250000	PRELSG_1258900	PF3D7_1339000	PRCDC_1338000	hypothetical protein
PGAL8A_00303400	PRELSG_1319300	PF3D7_1431800	PRCDC_1431100	apyrase, putative
PGAL8A_00233500	PRELSG_1206700	PF3D7_1474000	PRCDC_1473100	hypothetical protein
PGAL8A_00103400	PRELSG_0209700	PF3D7_0107300	PRCDC_0105200	hypothetical protein

Supplemental Table S3. Core genes specific to *P. knowlesi/P. vivax/P. malariae/P. ovale* clade and *P. gallinaceum, P. relictum*.

P. gallinaceum	*P. relictum*	*P. knowlesi H*	*P. vivax SalI*	*P. malariae*	*P. ovale curtisi*	Protein description
PGAL8A_00402200	PRELSG_0408500	PKNH_0409700	PVP01_0413400	PmUG01_04020800	PocGH01_04018600	WD repeat-containing protein, putative
PGAL8A_00405000	PRELSG_0405700	PKNH_0406500	PVP01_0410600	PmUG01_04018000	PocGH01_04015800	conserved Plasmodium protein, unknown function
PGAL8A_00089500	PRELSG_0511300	PKNH_0507700	PVP01_0522600	not present	PocGH01_05028200	hypothetical protein
PGAL8A_00443100	PRELSG_0727900	PKNH_0728800	PVP01_0728800	PmUG01_07041900	PocGH01_07037800	merozoite surface protein 1 paralog, putative (MSP1P)
PGAL8A_00072100	PRELSG_1006900	PKNH_1008000	PVP01_1009000	PmUG01_10019700	PocGH01_10016800	conserved Plasmodium protein, unknown function
PGAL8A_00039400	PRELSG_1014000	PKNH_1015400	PVP01_1016100	PmUG01_10026800	not present	transcription factor with AP2 domain(s), putative
PGAL8A_00237900	PRELSG_1202300	PKNH_1202800	PVP01_1228600	PmUG01_12038700	PocGH01_12036600	conserved Plasmodium protein, unknown function
PGAL8A_00214300	PRELSG_1225900	PKNH_1227000	PVP01_1251900	PmUG01_12061900	PocGH01_12059800	conserved Plasmodium protein, unknown function
PGAL8A_00311600	PRELSG_1102900	PKNH_1310400	PVP01_1309600	PmUG01_13020400	PocGH01_13020400	conserved Plasmodium protein, unknown function
PGAL8A_00466900	PRELSG_1461300	PKNH_1465900	PVP01_1463300	PmUG01_14079400	PocGH01_14070800	conserved Plasmodium protein, unknown function
PGAL8A_00277800	PRELSG_1344900	not present	PVP01_1342800	not present	not present	hypothetical protein
PGAL8A_00021800	PRELSG_0718600	not present	PVP01_0719500	not present	not present	WD repeat-containing protein, putative

Supplemental Table S4. Core genes pseudogenized in *P. gallinaceum* and/or *P. relictum*

Encoded proteins, or conceptual proteins from translated pseudogenes(*) are shown.

P. gallinaceum	*P. relictum*	Protein description
PGAL8A_00023600*	PRELSG_1030100	merozoite surface protein 8 (MSP8)
PGAL8A_00029100*	PRELSG_1024500	6-cysteine protein (P38)
PGAL8A_00151500*	PRELSG_1125300*	chorismate synthase (CS)
PGAL8A_00222900	PRELSG_1217300*	fam-a protein
PGAL8A_00358000	PRELSG_0928000*	methyltransferase
PGAL8A_00435300*	PRELSG_0720000*	para-aminobenzoic acid synthetase (pBAS)
PGAL8A_00476700	PRELSG_0601100*	conserved Plasmodium protein, unknown function
PGAL8A_00483100*	PRELSG_0607500	conserved Plasmodium protein, unknown function
PGAL8A_00065500	PRELSG_0028300*	multidrug resistance-associated protein

Supplemental Table S5. Core genes missing in *P. gallinaceum* and *P. relictum*

P. falciparum	*P. knowlesi*	*P. berghei*	*P. malariae*	Product
PF3D7_0206300	PKNH_0414600	PBANKA_0304000	PmUG01_04026100	pentafunctional AROM polypeptide
PF3D7_0710300	PKNH_0109000	PBANKA_1220900	PmUG01_01022100	conserved Plasmodium protein, unknown function
PF3D7_0821400	PKNH_1315400	PBANKA_0709400	PmUG01_05024700	conserved Plasmodium protein, unknown function
PF3D7_1006300	PKNH_0805100	PBANKA_1204500	PmUG01_08014200	conserved Plasmodium protein, unknown function

Supplemental Table S6. Multigene families in the genome of *P. gallinaceum* and *P. relictum.*
(The file is attached as file Supplemental_Table_S6)

Supplemental Table S7. Gene summary of *P. gallincaeum*
To each gene we include the functional annotation, number of exons, gene length and expression data (in RPKM). If available we further state the one to one orthologous to *P. relictum* and *P. falciparum*. Also reported is if a gene has Pexel, transmembrane domains, signal peptides or Pfam domains. (The file is attached as file Supplemental_Table_S7).

Supplemental Table S8. d_N/d_S values for all genes with 1:1 orthologous across six species.
4335 genes with 1:1 orthologues across *P. gallinaceum* (PGAL8A), *P. relictum* (PRELSG), *P. falciparum* (PF3D7), *P. reichenowi* (PRCDC), *P. knowlesi* (PKN) and *P. vivax* (PVX) are shown along with the annotation for the *P. falciparum* orthologue. For orthologues in each of the avian (PGAL8A vs PRELSG), falciparum (PF3D7 vs PRCDC) and vivax (PKN vs PVX) clades, d_N/d_S values were calculated and ranked. Gene expression data for *P. falciparum* 3D7 orthologues are from Lopez Barragán et al. 2011 (López-Barragán et al. 2011) and are shown as RPKM values, percentiles (from PlasmoDB), and the stage in which the gene is the 80th percentile of genes based on its high expression level. The annotation for *P. gallinaceum* 8A is from June 2017.

(The file is attached as file Supplemental_Table_S8).

Supplemental Table S9. Top 250 d_N/d_S values from pairwise comparisons within three lineages.
For all genes with 1:1 orthologues across *P. gallinaceum* (PGAL8A), *P. relictum* (PRELSG), *P. falciparum* (PF3D7), *P. reichenowi* (PRCDC), *P. knowlesi* (PKN) and *P. vivax* (PVX), d_N/d_S values were calculated and ranked in the avian (PGAL8A vs PRELSG), falciparum (PF3D7 vs PRCDC) and vivax (PKN vs PVX) clades. Only genes with an annotated function are listed. Quantiles of gene expression are from PlasmoDB (plasmodb.org) based on the source data from Lopez-Barragan (López-Barragán et al. 2011). 'Number of highly ranked lineages' refers to how many of the avian, falciparum and vivax lineages that a gene appears in the top 250 d_N/d_S values.

(The file is attached as file Supplemental_Table_S9).

Supplemental Table S10. d_N values for all genes with 1:1 orthologous across eight species.
4335 genes with 1:1 orthologues across *P. gallinaceum* (PGAL8A), *P. relictum* (PRELSG), *P. falciparum* (PF3D7), *P. reichenowi* (PRCDC), *P. knowlesi* (PKN), *P. vivax* (PVX), *P. berghei* (PBANKA) and *P. yoelii yoelii* (PY17X) are shown along with the annotation for the *P. falciparum* orthologue. For orthologues in each of the avian (PGAL8A vs PRELSG), falciparum (PF3D7 vs PRCDC) and vivax (PKN vs PVX) clades, d_N values were calculated and ranked. Gene expression data for *P. gallinaceum* orthologues are from Lopez Barragán (López-Barragán et al. 2011) and are shown as RPKM values, percentiles (from PlasmoDB), and the stage in which the gene is the 80th percentile of genes based

on its high expression level. The annotation for *P. gallinaceum* 8A is from June 2017. (The file is attached as file Supplemental_Table_S10).

Supplemental Methods

Relationship between *Plasmodium* species

The phylogenetic tree presented in this paper is robust to changes in the substitution model used for phylogenetic inference. The same phylogeny is from a maximum-likelihood tree under both partitioned and non-partitioned models (Supplemental Fig. S3A) and maximum parsimony analysis also agrees. Our result is also not just a result of trimming the alignment to remove poorly-aligned regions, as this tree also maximises the likelihood of a non-trimmed concatenated alignment under a simple substitution model. Simpler, non-model based approaches produce different trees, with neighbour-joining and simple amino acid distance producing a phylogeny matching that shown by Pick et al (Pick et al. 2011), with a clade of *Laverania* and avian malaria species, and the primate-infective species outside *Laverania* forming a clade related to a clade of rodent malaria (Supplemental Fig. S3B).

Whole genome sequencing of *P. gallinaceum*

From 20ng of the enriched genomic DNA whole genome amplification (WGA) was performed with REPLI-g Mini Kit (Qiagen) following a modified protocol (Oyola et al. 2014). Nuclease-free water and all tubes were UV-treated before use. WGA reactions were performed in 0.2 ml PCR tubes. Buffer D1 stock solution (Qiagen) was reconstituted by adding 500 µl of nuclease-free water and a working solution was prepared by mixing the stock solution and nuclease-free water in the ratio of 1:3.5 respectively. Buffer N1 was modified to include Tetramethylammonium chloride (TMAC) at a concentration of 300 mM. To denature DNA templates, 5 µl of the DNA solution was mixed with 5ul of buffer D1 (working solution prepared as described above). The mixture was vortexed and centrifuged briefly before incubating at room for 3 min. Denatured DNA was neutralized by adding 10 µl of the modified buffer N1. Neutralized DNA was mixed by vortexing and centrifuged briefly. To amplify the DNA template, denatured and neutralized sample was mixed with 29 µl of REPLI-g Mini Reaction Buffer and 1ul of REPLI-g Mini DNA polymerase to obtain a final reaction volume of 50 µl. The reaction mixture was incubated at 30°C for 16 hr using an MJ thermocycler with the heating lid set to track at +5°C. Amplified DNA was cleaned using Agencourt Ampure XP beads (Beckman Coulter) using sample to beads ration of 1:1 and eluted with 50 µl of EB (Qiagen).

Collection of genomic DNA from *P. relictum*

Heavily infected mosquito midguts were obtained from a laboratory line of *Cx. pipiens quinquefasciatus* (SLAB) that had been placed in a cage and allowed to blood feed from a heavily infected canary following standard laboratory protocols (Cornet et al. 2013). Two such cages, each with 70 mosquitoes, were set up in this way (bird parasitaemias were 4.45% and 7.89%). After the blood meal, mosquitoes were kept at 25°C and 80% relative humidity and dissected 7 days later to coincide with the midgut (oocyst) stage of the *Plasmodium* infection. Midguts were dissected and

oocyst numbers assessed using standard laboratory procedures (Zélé et al. 2014). Total DNA was extracted from a single pool of 50 heavily infected midguts (>100 oocysts) using the QIAGEN protocol and materials (DNeasy 96 Tissue Kit, Qiagen NV, Venlo, The Netherlands) and total DNA was eluted in the final step with 100µL RNase free water (Qiagen).

Genome assembly of *P. relictum*

Low quality regions of sequencing reads were clipped with SGA version 0.9.1 (Simpson and Durbin 2012; parameters: -m 51 --permute-ambiguous -f 3 -q 3) and assembled with Velvet (version 1.2.07) (Zerbino and Birney 2008), using a k-mer of 81 selected by iterative testing (k-mers: 85, 81, 71 and 55). The other parameters were: -exp_cov 17 -max_coverage 30 -ins_length 450 -ins_length_sd 30 -cov_cutoff 9 -min_contig_lgth 200 -min_pair_count 10. Contigs were further scaffolded with SSPACE (Boetzer et al. 2011). The assembly was improved as described in PAGIT (Post Assembly Genome Improvement Toolkit (Swain et al. 2012). First, contigs were ordered with with ABACAS (Assefa et al. 2009) against *P. knowlesi*. Several rounds of iCORN2 (Otto et al. 2010) corrected single base pair errors and small indels. Assembly errors were detected with REAPR (Hunt et al. 2013), and contigs broken contig at each Fragment Coverage Distribution error (Parameter -l to also break contig errors). Those corrected contigs were ordered again with respect to described reference genomes. Next, sequencing gaps were further closed with GapFiller (Boetzer and Pirovano 2012) and six iterations with IMAGE (Tsai et al. 2010), with two iterations of each of the decreasing k-mer lengths 71, 55 and 41.

Phylogenetic analysis

OrthoMCL v2.0 was used to cluster predicted proteins from 19 species of *Apicomplexan* parasites, including 11 previously published *Plasmodium* species: **P. berghei*, **P. chabaudi*, **P. yoelli, *P. cynomolgi, *P. falciparum*, **P. knowlesi* (Pain et al. 2008), **P. reichenowi*, **P. vivax, †P. ovale and †P. malariae*, the published *Haemoproteus tartakovskysi* genome (Bensch et al. 2016) and the two new *Plasmodium* genomes described here, together with *Toxoplasma gondii* and the piroplasms *Babesia microti, Babesia bovis, Theileria parvum* and *Theileria annulata*. Data for published *Plasmodium* genomes were downloaded from GeneDB (http://www.genedb.org; Logan-Klumpler et al. 2012) on 17/7/2013 (species marked * above) or 01/06/2016 (species marked †). Data for non-*Plasmodium* species were downloaded from apiDB (http://www.apidb.org) on 01/06/2016. OrthoMCL was run with default parameters and an inflation parameter of 1.5. The output was parsed to identify a total of 881 clusters that were single-copy and present in all 19 species. Amino acid sequences for all of these clusters were aligned using mafft v7.205 (Katoh and Standley 2013) with the '--auto' flag and other parameters as defaults. These alignments were trimmed using GBlocks v0.91b (Castresana 2000) to keep well-aligned blocks of at least four consecutive well-aligned columns, separated by up to four less-conserved columns, and to discard columns with gap characters in at least 50% of sequences. All trimmed gene cluster alignments with more than 10 amino acid residues (879 out of 881) were kept for subsequent analysis. Subsequent phylogenetic analyses were all based on this alignment of 289,315 amino acid residues, from 879 single-copy gene clusters.

Bayesian phylogenetic inference was performed using PhyloBayes 3.3f (Lartillot et al. 2009) under a CAT mixture model, allowing the rate of substitutions to vary between sites according to a discretised gamma distribution and the substitution process at each site to come from a mixture of

amino acid composition matrices but with a single underlying Poisson process for the substitution process. We ran 8 independent MCMC chains of at least 60,000 steps each. The final 1500 trees from each chain were concatenated for inference (discarding approximately 20,000 steps per chain as burn-in). While model parameter estimates had not all converged across all chains, tree topologies appeared to be following visualisation with "R We There Yet?" (https://github.com/danlwarren/RWTY). Maximum-likelihood phylogenetic analysis using RAxML v.8.0.24 (Stamatakis 2014) was performed using a partitioned model where the alignment for each locus was assigned the best-fitting model under BIC from the set of empirical amino acid substitution matrices available in that version of RAxML and using observed amino acid composition, and under a single LG4X model for the whole alignment with maximum-likelihood estimates of amino acid composition. Additional analyses used PAUP v4.0b10 and Phylip v3.6.9 (Felsenstein 2005) for parsimony and neighbour-joining analysis of standard AA pairwise distances (under the JTT model) and Log-Det distances calculated using LDDist v1.3.2 (Thollesson 2004).

To generate the RBP and transposable element (TE) trees, we trimmed the alignments with Gblocks in Seaview version 4.3.1 (Galtier et al. 1996) allowing the loosest settings. The models were estimated with RAXML and 100 bootstraps. The models PROTGAMMALG4M and PROTGAMMAGTR were used for the TE and the RPB analyses, respectively. To select the sequence for the TE tree, we BLAST-searched the *P. gallinaceum* TE against the non-redundant nucleotide database, took all the hits and included the TE sequences from *Eimeria* and yeast (U6KAF4_9EIME, U6GBW4_9EIME and YG31B_YEAST). Using a simple randomisation approach the association of GC content with distinct TE clades was tested. 10,000 sets of random GC content were constructed with the same size as each of the four main TE clades and the frequency in which the observed GC partitioning was reproduced. For both *P. gallinaceum* clades, the results were significant (p ~= 0.0059 for the big clade of higher mean percentage GC and $p < 0.0001$ for the smaller clade of lower mean percentage GC).

Dating analysis

Using the Bayesian coalescence method G-PhoCS (Gronau et al. 2011) and several genotypes, the divergence times of *P. malariae* and *P. malariae-like* have previously been estimated to be similar to that of *P. falciparum* and the chimpanzee parasite *P. reichenowi* (Rutledge et al. 2017). Using the same method, the divergence of *Plasmodium ovale wallikeri* and *P. o. curtisi* was estimated to have occurred 5 times earlier. By analyzing mutation rates and in vivo data, the divergence of *P. falciparum* and *P. reichenowi* has been estimated to have occurred approximately 200,000 years ago (Otto et al. 2017) and the *P. ovale* split must have therefore occurred around 1 million years ago. This is a revised estimate from Rutledge *et al* (Rutledge et al. 2017) where the date of the *P. ovale* split had been calibrated on previously published estimates for the *P. reichenowi-P.falciparum* (3.5 - 5.5 MYA) split.

With only a single representative sample for each avian-infective species, it is not possible to use G-PhoCS to estimate divergence times but the dates for other species dates can be used as guides.

We used a method based on a Total Least Squares regression and the existence of a molecular clock specific to *Plasmodium* (Silva et al. 2015) to estimate speciation dates. To implement and test the method, we first generated amino acid alignments of 18 species from 2,915 one-to-one orthologues.

This set included species whose speciation times have been dated using coalescence modelling (Rutledge et al. 2017; Otto et al. 2017). Different to the original work of Silva *et al*. (Silva et al. 2015) we generated an alignment including all the protein sequence of all the 18 species, rather than pairwise comparisons – which is now possible due to better genome sequences, for example for *P. reichenowi* (Otto et al. 2014b) that had just around 445 one-to-one orthologues in the original work. The alignments for each orthologous group were performed with mafft (--auto parameter), and further trimmed with Gblocks (Talavera and Castresana 2007) (parameter -t=p --b5=h -p=n -b4=2) to exclude gaps and badly aligned regions. Following the original work, we obtained the control file with PAML (Yang 2007) and the R code from the author for the Total Least Squares regression.

First, the known speciation timings were evaluated. As expected, the speciation of *P. ovale walikeri* and *P. o. curtsi* was predicted to be 5x earlier than the *P. malariae* and *P. malariae-like* split, confirming the validity of the method. Further, the relative time between the split of *P. reichenowi* with *P. falciparum* and *P. praefalciparum* with *P. falciparum* was predicted as in (Otto et al. 2017). In contrast, the relative timing of *P. malariae* separating from *P. malariae-like* and *P. reichenowi* from *P. falciparum* was predicted to be 2.5x apart. This apparent discrepancy is probably due to the huge difference in GC content and the resulting amino acid bias that influences the molecular clock.

Next the method was applied to all orthologous core genes (Figure 2) but with the 250 genes with the highest d_N values excluded, as non-neutrally evolving outliers, resulting in 3646 orthologues. Using previous estimates (Otto et al. 2017), the *P. ovale* split can be calibrated to 1 million years ago. The divergence of the avian and mammalian *Plasmodium* lineages therefore occurred between 10-13 million years ago (Figure 2). The *P. ovale* split has previously been estimated to be around 3 million years ago (Sutherland et al. 2010), resulting in an estimate of 30-40 million years ago for the split of avian and mammalian *Plasmodium*, still an order of magnitude more recent than the estimated dates for the avian and mammal lineages diverging around 320 million years ago (Kumar and Hedges 1998).

Supplemental References

Assefa S, Keane TM, Otto TD, Newbold C, Berriman M. 2009. ABACAS: algorithm-based automatic contiguation of assembled sequences. *Bioinforma Oxf Engl* **25**: 1968–1969.

Bensch S, Canbäck B, DeBarry JD, Johansson T, Hellgren O, Kissinger JC, Palinauskas V, Videvall E, Valkiūnas G. 2016. The Genome of Haemoproteus tartakovskyi and Its Relationship to Human Malaria Parasites. *Genome Biol Evol* **8**: 1361–1373.

Boetzer M, Henkel CV, Jansen HJ, Butler D, Pirovano W. 2011. Scaffolding pre-assembled contigs using SSPACE. *Bioinforma Oxf Engl* **27**: 578–579.

Boetzer M, Pirovano W. 2012. Toward almost closed genomes with GapFiller. *Genome Biol* **13**: R56.

Castresana J. 2000. Selection of conserved blocks from multiple alignments for their use in phylogenetic analysis. *Mol Biol Evol* **17**: 540–552.

Cornet S, Nicot A, Rivero A, Gandon S. 2013. Malaria infection increases bird attractiveness to uninfected mosquitoes. *Ecol Lett* **16**: 323–329.

Felsenstein J. 2005. PHYLIP (Phylogeny Inference Package) version 3.6. *Distrib Author*.

Galtier N, Gouy M, Gautier C. 1996. SEAVIEW and PHYLO_WIN: two graphic tools for sequence alignment and molecular phylogeny. *Comput Appl Biosci CABIOS* **12**: 543–548.

Ginsburg H, Abdel-Haleem AM. 2016. Malaria Parasite Metabolic Pathways (MPMP) Upgraded with Targeted Chemical Compounds. *Trends Parasitol* **32**: 7–9.

Gronau I, Hubisz MJ, Gulko B, Danko CG, Siepel A. 2011. Bayesian inference of ancient human demography from individual genome sequences. *Nat Genet* **43**: 1031–1034.

Hunt M, Kikuchi T, Sanders M, Newbold C, Berriman M, Otto TD. 2013. REAPR: a universal tool for genome assembly evaluation. *Genome Biol* **14**: R47.

Katoh K, Standley DM. 2013. MAFFT multiple sequence alignment software version 7: improvements in performance and usability. *Mol Biol Evol* **30**: 772–780.

Kumar S, Hedges SB. 1998. A molecular timescale for vertebrate evolution. *Nature* **392**: 917–920.

Lartillot N, Lepage T, Blanquart S. 2009. PhyloBayes 3: a Bayesian software package for phylogenetic reconstruction and molecular dating. *Bioinforma Oxf Engl* **25**: 2286–2288.

Logan-Klumpler FJ, De Silva N, Boehme U, Rogers MB, Velarde G, McQuillan JA, Carver T, Aslett M, Olsen C, Subramanian S, et al. 2012. GeneDB--an annotation database for pathogens. *Nucleic Acids Res* **40**: D98-108.

López-Barragán MJ, Lemieux J, Quiñones M, Williamson KC, Molina-Cruz A, Cui K, Barillas-Mury C, Zhao K, Su X. 2011. Directional gene expression and antisense transcripts in sexual and asexual stages of Plasmodium falciparum. *BMC Genomics* **12**: 587.

Otto TD, Gilabert A, Crellen T, Böhme U, Arnathau C, Sanders M, Oyola S, Okauga AP, Boundenga L, Wuillaume E, et al. 2017. Genomes of an entire Plasmodium subgenus reveal paths to virulent human malaria. bioRxiv doi: https://doi.org/10.1101/095679.

Otto TD, Rayner JC, Böhme U, Pain A, Spottiswoode N, Sanders M, Quail M, Ollomo B, Renaud F, Thomas AW, et al. 2014. Genome sequencing of chimpanzee malaria parasites reveals possible pathways of adaptation to human hosts. *Nat Commun* **5**: 4754.

Otto TD, Sanders M, Berriman M, Newbold C. 2010. Iterative Correction of Reference Nucleotides (iCORN) using second generation sequencing technology. *Bioinforma Oxf Engl* **26**: 1704–1707.

Oyola SO, Manske M, Campino S, Claessens A, Hamilton WL, Kekre M, Drury E, Mead D, Gu Y, Miles A, et al. 2014. Optimized whole-genome amplification strategy for extremely AT-biased template. *DNA Res Int J Rapid Publ Rep Genes Genomes* **21**: 661–671.

Pain A, Böhme U, Berry AE, Mungall K, Finn RD, Jackson AP, Mourier T, Mistry J, Pasini EM, Aslett MA, et al. 2008. The genome of the simian and human malaria parasite Plasmodium knowlesi. *Nature* **455**: 799–803.

Pick C, Ebersberger I, Spielmann T, Bruchhaus I, Burmester T. 2011. Phylogenomic analyses of malaria parasites and evolution of their exported proteins. *BMC Evol Biol* **11**: 167.

Rutledge GG, Böhme U, Sanders M, Reid AJ, Cotton JA, Maiga-Ascofare O, Djimdé AA, Apinjoh TO, Amenga-Etego L, Manske M, et al. 2017. Plasmodium malariae and P. ovale genomes provide insights into malaria parasite evolution. *Nature* **542**: 101–104.

Silva JC, Egan A, Arze C, Spouge JL, Harris DG. 2015. A New Method for Estimating Species Age Supports the Coexistence of Malaria Parasites and Their Mammalian Hosts. *Mol Biol Evol* **32**: 1354–1364.

Simpson JT, Durbin R. 2012. Efficient de novo assembly of large genomes using compressed data structures. *Genome Res* **22**: 549–556.

Stamatakis A. 2014. RAxML version 8: a tool for phylogenetic analysis and post-analysis of large phylogenies. *Bioinforma Oxf Engl* **30**: 1312–1313.

Sutherland CJ, Tanomsing N, Nolder D, Oguike M, Jennison C, Pukrittayakamee S, Dolecek C, Hien TT, do Rosário VE, Arez AP, et al. 2010. Two nonrecombining sympatric forms of the human malaria parasite Plasmodium ovale occur globally. *J Infect Dis* **201**: 1544–1550.

Swain MT, Tsai IJ, Assefa SA, Newbold C, Berriman M, Otto TD. 2012. A post-assembly genome-improvement toolkit (PAGIT) to obtain annotated genomes from contigs. *Nat Protoc* **7**: 1260–1284.

Talavera G, Castresana J. 2007. Improvement of phylogenies after removing divergent and ambiguously aligned blocks from protein sequence alignments. *Syst Biol* **56**: 564–577.

Thollesson M. 2004. LDDist: a Perl module for calculating LogDet pair-wise distances for protein and nucleotide sequences. *Bioinforma Oxf Engl* **20**: 416–418.

Tsai IJ, Otto TD, Berriman M. 2010. Improving draft assemblies by iterative mapping and assembly of short reads to eliminate gaps. *Genome Biol* **11**: R41.

Yang J, Yan R, Roy A, Xu D, Poisson J, Zhang Y. 2015. The I-TASSER Suite: protein structure and function prediction. *Nat Methods* **12**: 7–8.

Yang Z. 2007. PAML 4: phylogenetic analysis by maximum likelihood. *Mol Biol Evol* **24**: 1586–1591.

Zélé F, Nicot A, Berthomieu A, Weill M, Duron O, Rivero A. 2014. Wolbachia increases susceptibility to Plasmodium infection in a natural system. *Proc Biol Sci* **281**: 20132837.

Zerbino DR, Birney E. 2008. Velvet: algorithms for de novo short read assembly using de Bruijn graphs. *Genome Res* **18**: 821–829.
